# The brain transcriptome of the wolf spider, *Schizocosa ocreata*

**DOI:** 10.1186/s13104-021-05648-y

**Published:** 2021-06-23

**Authors:** Daniel Stribling, Peter L. Chang, Justin E. Dalton, Christopher A. Conow, Malcolm Rosenthal, Eileen Hebets, Rita M. Graze, Michelle N. Arbeitman

**Affiliations:** 1 Biomedical Sciences Department, College of Medicine, Florida State University, Tallahassee, FL 32306 USA; 2 Department of Biological Sciences, University of Southern California, Los Angeles, CA 90089 USA; 3 School of Biological Sciences, University of Nebraska-Lincoln, Lincoln, NE 68588 USA; 4 Department of Biological Sciences, Auburn University, Auburn, AL 36849 USA; 5 Present Address: Department of Molecular Genetics and Microbiology, Genetics Institute, University of Florida, Gainesville, FL 32610 USA

**Keywords:** *Schizocosa ocreata*, Wolf spiders, Gene expression, Transcriptomes, Sexual dimorphism, Sex-differential gene expression, Sex biased expression, Brain and central nervous system, De novo transcriptome assembly

## Abstract

**Objectives:**

Arachnids have fascinating and unique biology, particularly for questions on sex differences and behavior, creating the potential for development of powerful emerging models in this group. Recent advances in genomic techniques have paved the way for a significant increase in the breadth of genomic studies in non-model organisms. One growing area of research is comparative transcriptomics. When phylogenetic relationships to model organisms are known, comparative genomic studies provide context for analysis of homologous genes and pathways. The goal of this study was to lay the groundwork for comparative transcriptomics of sex differences in the brain of wolf spiders, a non-model organism of the pyhlum Euarthropoda, by generating transcriptomes and analyzing gene expression.

**Data description:**

To examine sex-differential gene expression, short read transcript sequencing and de novo transcriptome assembly were performed. Messenger RNA was isolated from brain tissue of male and female subadult and mature wolf spiders (*Schizocosa ocreata*). The raw data consist of sequences for the two different life stages in each sex. Computational analyses on these data include de novo transcriptome assembly and differential expression analyses. Sample-specific and combined transcriptomes, gene annotations, and differential expression results are described in this data note and are available from publicly-available databases.

## Objectives

Arachnids, including spiders, have diverse and unique reproductive behavior, including sexual cannibalism and female aggression, copulatory wounding, and elaborate courtship with sexual dimorphism in morphology and coloration [1–5]. The development of genomic resources in arachnids will allow for key comparisons not only in genome biology, but also in evolution and in the biology of sex. Comparative studies between arachnids and model organisms in other arthropod classes can provide a broader set of inferences that goes beyond what has been learned from model organisms. For example, copulatory wounding, male leg ornamentation, and elaborate courtship are well-studied in Drosophila, and arachnid genomic comparative studies can reveal parallel or divergent mechanisms [6–15].

Several arachnid genomes and transcriptomes, including those of spiders, mites and scorpions, have recently become available [16–18]. Given that spiders have unique sex-specific behaviors and that progress is ongoing in developing arachnid genomics, our goal was to generate transcriptomes and gene expression data using mRNA from brains of males and females of the wolf spider, *Schizocosa ocreata*. Studies of wolf spider sexual dimorphism in morphology and behavior have revealed intriguing parallels to textbook examples of sex dimorphism in well-studied model organisms [19–25]. The data presented here are valuable in laying necessary groundwork for broad comparative functional genomics of sex differences in brain and behavior across arthropods.

## Data description

mRNA was isolated from brain samples of immature (Imm; subadult) and mature (Mat; adult) male and female *Schizocosa ocreata*, collected in Lancaster County, Nebraska (see detailed methods: NCBI Gene Expression Omnibus [GEO] Series accession number GSE168766). Illumina paired-end sequencing was performed with libraries generated from mRNA derived from individual brain samples, with three replicates for each sex/stage (Data set 1; NCBI SRA: SRP302932). Sequence reads were processed to remove index and low-quality sequences; quality assessments are provided (Data file 1; Table 1; GEO GSE168766).

**Table 1:** Overview of data files/data sets

Label	Name of data file/data set	File types (file extension)	Data repository and identifier (DOI or accession number)
Data set 1	Raw illumina data	FASTQ files (.fq)	NCBI SRA: https://identifiers.org/ncbi/insdc.sra:SRP302932 [45]
Data file 1	Trimmed-read FastQC statistics	PDF file (.pdf)	NCBI GEO: https://identifiers.org/geo:GSE168766 [46]
Data set 2	*Schizocosa ocreata* immature male 1, de novo transcriptome assembly	FASTA files (.fa)	NCBI TSA: https://identifiers.org/ncbi/insdc:GIZN00000000 [47]
Data set 3	*Schizocosa ocreata* immature male 2, de novo transcriptome assembly	FASTA files (.fa)	NCBI TSA: https://identifiers.org/ncbi/insdc:GIZS00000000 [48]
Data set 4	*Schizocosa ocreata* immature male 3, de novo transcriptome assembly	FASTA files (.fa)	NCBI TSA: https://identifiers.org/ncbi/insdc:GIZT00000000 [49]
Data set 5	*Schizocosa ocreata* immature female 2, de novo transcriptome assembly	FASTA files (.fa)	NCBI TSA: https://identifiers.org/ncbi/insdc:GIZM00000000 [50]
Data set 6	*Schizocosa ocreata* immature female 3, de novo transcriptome assembly	FASTA files (.fa)	NCBI TSA: https://identifiers.org/ncbi/insdc:GIZR00000000 [51]
Data set 7	*Schizocosa ocreata* mature male 2, de novo transcriptome assembly	FASTA files (.fa)	NCBI TSA: https://identifiers.org/ncbi/insdc:GIZP00000000 [52]
Data set 8	*Schizocosa ocreata* mature male 3, de novo transcriptome assembly	FASTA files (.fa)	NCBI TSA: https://identifiers.org/ncbi/insdc:GIZW00000000 [53]
Data set 9	*Schizocosa ocreata* mature female 1, de novo transcriptome assembly	FASTA files (.fa)	NCBI TSA: https://identifiers.org/ncbi/insdc:GIZO00000000 [54]
Data set 10	*Schizocosa ocreata* mature female 2, de novo transcriptome assembly	FASTA files (.fa)	NCBI TSA: https://identifiers.org/ncbi/insdc:GIZU00000000 [55]
Data set 11	*Schizocosa ocreata* mature female 3, de novo transcriptome assembly	FASTA files (.fa)	NCBI TSA: https://identifiers.org/ncbi/insdc:GIZV00000000 [56]
Data set 12	*Schizocosa ocreata* consensus coding sequences	FASTA file (.fa)	NCBI TSA: https://identifiers.org/ncbi/insdc:GIZQ00000000 [57]
Data file 2	Transcriptome assembly statistics	PDF file (.pdf)	NCBI GEO https://identifiers.org/geo:GSE168766 [46]
Data file 3	Gene annotations	Tabular text file (.txt)	NCBI GEO https://identifiers.org/geo:GSE168766 [46]
Data file 4	Gene expression values	Tabular text file (.txt)	NCBI GEO https://identifiers.org/geo:GSE168766 [46]
Data file 5	Differential expression values	MS excel file (.xlsx)	NCBI GEO https://identifiers.org/geo:GSE168766 [46]
Data file 6	Transcriptome flow (TFLOW): de novo transcriptome analysis pipeline	Zip archive (.zip)	Zenodo 10.5281/zenodo.3817474 [58]
Data file 7	Gene clustering analysis script	Python script (.py)	Zenodo 10.5281/zenodo.4330738 [59]
Data file 8	Differential expression analysis script	R script (.R)	Zenodo 10.5281/zenodo.4330738 [59]

Transcriptome assembly was performed for each sample using Trinity followed by CAP3 [26, 27]. A consensus transcriptome was assembled combining all individual assemblies using CAP3. Transcriptome quality was evaluated on individual sample and consensus transcriptome assemblies based on the number of conserved protein coding genes identified from the Core Eukaryotic Genes Mapping Approach (CEGMA) and Benchmarking sets of Universal Single-Copy Orthologs (BUSCO) Arthropod databases (Data file 2; GEO GSE168766) [28–30]. Both CEGMA and BUSCO alignments used the Basic Local Alignment Search (BLAST) utility with default threshold E-value of 1e−20 [31]. In the consensus assembly, 99% of CEGMA and 95% of BUSCO genes were identified, demonstrating a high assembly quality. To facilitate this workflow, the Transcriptome-Flow (TFLOW) pipeline was developed (Python 2.7; Zenodo; Data file 6). Each individual assembly was filtered to remove contaminant sequences and uploaded to the NCBI Transcriptome Shotgun Assembly (TSA) database (Data sets 2–11; Table 1).

Putative protein coding sequences from the consensus assembly were extracted using TransDecoder v5.3.0 [32]. Coding sequences were annotated using Trinonate [33], aligning coding sequence (CDS) and predicted protein sequences against several databases, including Uniprot (October 2018), NCBI nr, and the Flybase *Drosophila melanogaster* v6.23 draft genome (Data file 3; GEO GSE168766). These alignments were used for identification of Protein Family (Pfam) domains, Gene Ontology (GO), and Kyoto Encyclopedia of Genes and Genomes (KEGG) identifiers [34–39].

Annotated coding sequences were clustered into genes based on sequence similarity determined by an all-vs-all BLASTn analysis, with software archived on Zenodo (Data file 7). Sequences identified as contaminants were removed and consensus CDS sequences were uploaded to the TSA database (Data set 12: TSA GIZQ00000000). For analysis of differential expression, reads were aligned to consensus CDS sequences and assigned to gene clusters, with expression estimated as read counts per gene (Data file 4; GEO GSE168766). Read alignment was performed using Burrows–Wheeler Aligner (BWA-MEM, version 0.6.1-r104) [40, 41]. A linear model was fit with the glmLRT function in edgeR (version 3.1.2) using default (trimmed mean of M values, TMM) normalization [42–44]. Likelihood ratio tests were constructed with comparisons between: (1) immature vs mature adult data within sex; (2) all immature vs all adult data from both sexes; (3) male vs. female data at each stage; and (4) all male vs all female data from both stages. The calculated log fold-change (logFC), log counts-per-million (logCPM), Likelihood-ratio (LR), p-value, and false discovery rate (FDR) adjusted p-value are reported (Data file 5; GEO GSE168766). The R-script has been archived on Zenodo (Data file 8).

## Limitations

Following read processing and quality assessment, two libraries (immature female 1 and adult male 1) were excluded from further analysis due to low sequence coverage. This limits the power to detect differential expression in the corresponding comparisons. The quality of the transcriptomes could be improved by coupling these data with long-read sequencing data in future work. Since the completion of this study, the CEGMA annotation database has been discontinued. The TFLOW software package was developed in the Python2.7 programming language which is no longer actively supported. Archival versions of Python2.7 may be utilized to execute TFLOW, or conversion of this software to a currently-supported version of Python can be performed using a python version-update package.

## Data Availability

The data described in this Data note can be freely and openly accessed on the NCBI SRA database under accession number SRP302932 (https://identifiers.org/ncbi/insdc.sra:SRP302932); the NCBI TSA database under BioProject accession number PRJNA694130 (https://identifiers.org/bioproject:PRJNA694130; full accession list in Table 1); and the NCBI GEO database under accession number GSE168766 (https://identifiers.org/geo:GSE168766) [45–57]. The computational pipeline utilized for transcriptome assembly and quality assessment is available through Zenodo under DOI: https://doi.org/10.5281/zenodo.3817475 [58]. The scripts utilized for clustering and differential expression analysis are available through Zenodo under DOI: 10.5281/zenodo.4330738 [59]. Please see Table 1 and references [45–59] for details and links to the data.

## Abbreviations

CEGMA: Core Eukaryotic Genes Mapping Approach
BUSCO: Benchmarking sets of Universal Single-Copy Orthologs
BLAST: Basic Local Alignment Search Tool
BWA: Burrows–Wheeler Aligner
CDS: Coding sequence
FDR: False discovery rate
GO: Gene Ontology
GEO: Gene Expression Omnibus
Imm: Immature
KEGG: Kyoto Encyclopedia of Genes and Genomes
logCPM: Log counts-per-million
logFC: Log fold-change
LR: Likelihood-ratio
Mat: Mature
mRNA: Messenger RNA
NCBI: National Center for Biotechnology Information
Pfam: Protein family
TMM: Trimmed mean of M-value
RNA-seq: RNA sequencing
TSA: Transcriptome Shotgun Assembly
SRA: Sequence Read Archive
